# P-1941. COVID-19 Impact on Acute Pancreatitis Hospitalization and Mortality: A Retrospective United States Cohort Study

**DOI:** 10.1093/ofid/ofae631.2100

**Published:** 2025-01-29

**Authors:** Thanathip Suenghataiphorn, Tuntanut Lohawatcharagul, Phuuwadith Wattanachayakul, Narathorn Kulthamrongsri, Pojsakorn Danpanichkul, Natchaya Polpichai, Jerapas Thongpiya

**Affiliations:** Griffin Hospital, Ansonia, Connecticut; Faculty of Medicine, Chulalongkorn University and King Chulalongkorn Memorial Hospital, Bangkok, Krung Thep, Thailand; Albert Einstein Healthcare Network, Philadelphia, Pennsylvania; Mayo Clinic, Pheonix, Arizona; Texas Tech University Health Science Center, Lubbock, Texas; Weiss Memorial Hospital, Chicago, Illinois; Texas Tech University Health Science Center, Lubbock, Texas

## Abstract

**Background:**

Acute pancreatitis, a prevalent gastrointestinal condition, frequently necessitates hospitalization and is associated with potential adverse in-hospital outcomes. Recent data showed the detrimental effects of COVID-19 infection on acute pancreatitis, particularly in patients with severe COVID-19 infections. However, limited information exists on the specific impacts of COVID-19 infection in patients hospitalized for acute pancreatitis.

**Methods:**

We analyzed the 2020 U.S. National Inpatient Sample (NIS) to investigate the effects of COVID-19 infection on cases primarily admitted due to acute pancreatitis. Participants aged 18 and above were identified using relevant ICD-10 CM codes. Adjusted odds ratios (aORs) for specified outcomes were calculated through multivariable logistic and linear regression analyses. The primary outcome was inpatient mortality, with secondary outcomes including system-based complications. Statistical significance was established at a P value of 0.05.

**Results:**

We identified 252,435 patients with a primary discharge diagnosis of acute pancreatitis. The mean age was 50.1 years; 48.6% were female. Caucasians accounted for 41%, followed by Hispanics. Of these, 1.05% (2,675/252,435) had a concurrent diagnosis of COVID-19 infection.

In a survey multivariable logistic and linear regression model adjusting for patient and hospital factors, COVID-19 infection was associated with higher in-hospital mortality (aOR 3.71; 95% CI 2.01, 6.84, P < 0.001), prolonged length of stay (β LOS 1.05; 95% CI 0.46, 1.64, P < 0.001), increased hospitalization charge ( β charge 9,327; 95% CI 820, 17834, P < 0.001), and acute respiratory failure (aOR 2.49; 95% CI 1.75, 3.53, P < 0.001), We observed non-significant, but increased, risks of shock, acute kidney injury (AKI), pancreatic pseudocyst, acute respiratory distress syndrome (ARDS), and mechanical ventilation in COVID-19 positive patients.

**Conclusion:**

In conclusion, our study underscores that COVID-19 infection is associated with higher in-hospital mortality, prolonged hospital stays, and increased incidence of acute respiratory failure in patients diagnosed with acute pancreatitis. Future longitudinal studies are warranted to comprehensively assess the long-term health sequelae in this population.

**Disclosures:**

All Authors: No reported disclosures

